# Changes in Root–Shoot Allometric Relations in Alpine Norway Spruce Trees After Strip Cutting

**DOI:** 10.3389/fpls.2021.703674

**Published:** 2021-08-27

**Authors:** Petia Simeonova Nikolova, Jan Geyer, Peter Brang, Paolo Cherubini, Stephan Zimmermann, Holger Gärtner

**Affiliations:** ^1^Forest Resources and Management, Swiss Federal Research Institute WSL, Birmensdorf, Switzerland; ^2^Forest Dynamics, Swiss Federal Research Institute WSL, Birmensdorf, Switzerland; ^3^Department of Forest and Conservation Sciences, Faculty of Forestry, University of British Columbia, Vancouver, BC, Canada; ^4^Forest Soils and Biogeochemistry, Swiss Federal Research Institute WSL, Birmensdorf, Switzerland

**Keywords:** strip cutting, mountain forests, Norway spruce, edge trees, biomass allocation, root-shoot allometric responses, tree size

## Abstract

Silvicultural interventions such as strip cuttings can change the resource availability of the edge trees. This may alter tree allometry, as light regime, water, and nutrient availability can change at the forest edge. Increased root growth may optimize resource uptake and/or enhance tree anchorage to withstand the altered wind regime. However, little is known about the patterns of the root–shoot allometric responses to strip cuttings. In three alpine stands differing in climate, site productivity, and stand characteristics, we selected 71 Norway spruce trees and took increment cores from stems, root collars, and main roots. This enabled us to study changes in the long-term root-stem allometry for 46 years and short-term allometric responses to intervention. The effects of cutting were compared between edge trees and trees from the stand interior in 10 years before and after the intervention. The long-term allocation to roots increased with stem diameter, with the strongest effects on the regularly managed stand with the tallest and largest trees. These results support the allometric biomass partitioning theory, which postulates resource allocation patterns between different plant organs to depend on plant size. Strip cutting on north-facing slopes boosted edge-tree growth in all plant compartments and enhanced allocation to roots. This change in allometry started 2 years after cutting but disappeared 7–8 years later. In the post-cutting period, the highest root–shoot increase was observed in the small trees independent of the site. This indicates the change in growing conditions to have the strongest effects in formerly suppressed trees. Thus, the effect of such acclimation on the wind firmness of subdominant spruce trees is a question with high importance for optimizing cutting layouts in lowering post-cutting vulnerability to disturbance. The results from this case study contribute to a better understanding of the structural acclimation of spruce trees from high-elevation forests to new forest edges. However, for a more mechanistic understanding of environmental drivers, further analyses of tree-ring stable isotopes are recommended.

## Introduction

Investigations about growth allocation patterns in trees are fundamental to select the appropriate silvicultural treatments to improve the growth of economically important plant organs (Poorter and Sack, 2012; Poorter et al., 2012) and to understand the extent to which trees or tree stands are susceptible to disturbances (Wonn and O'Hara, 2001; Pretzsch et al., 2014). Changes in relative growth or size of different plant parts are the focus of allometric studies (Huxley and Tessier, 1936; Niklas, 2005) with root–shoot development being the best-analyzed allometric relationship (Shipley and Meziane, 2002; Mokany et al., 2006). The ability of a tree to shift biomass growth toward above- or belowground structures is a key trait for adapting to changing site conditions (Bolte et al., 2004; Dumroese et al., 2019) and depends on (1) environmental effects (e.g., duration and magnitude of changes in resource availability; Weinstein et al., 1991; Callaway et al., 1994), (2) phylogeny (e.g., differences between deciduous and evergreen species; Drexhage and Colin, 2001; Poorter et al., 2012), and (3) ontogeny (i.e., resource allocation patterns change with plant size; Shipley and Meziane, 2002; Niklas, 2005).

In forest stands, the allocation patterns of individual trees can be altered by management (Pretzsch et al., 2014). For example, the competition release induced by thinning may boost root expansion since more soil water becomes available to cover, among others, the increased transpiration demand for tree growth (Tyree, 2003). An overproportional belowground growth enhances the weight of the root-soil plate itself, which is an important structural component of tree anchorage and resistance to uprooting (Ennos, 1993). An altered wind regime due to cutting can also alter cambial growth as a direct response to tree movement, with accelerated growth close to the stem base (in the zone of the root collar), as compared to the upper stem parts (Nicoll and Ray, 1996; Nicoll and Dunn, 2000). However, quantitative information on these aspects is still scarce. Failure to understand how trees adapt structurally and functionally to competition-release may lead to erroneous conclusions about tree vigor and mechanical stability in response to disturbance and management.

For many conifers that tend to develop shallow and plate-formed root systems (Stokes and Mattheck, 1996; Polomski and Kuhn, 2001), the ability to redistribute resources to roots seems essential in adapting to imposed mechanical stress (Urban et al., 1994; Telewski, 1995; Stokes, 2002). For example, in a balsam fir-black spruce forest in Québec (Canada), retention cutting induced faster roots than stem growth (Pretzsch et al., 2014). The interpretation for the observed change in growth allocation was that the newly exposed trees needed to increase their mechanical stability against windthrow, by biomass allocation to the root system for stronger anchorage (see also Nielsen, 1995). Another study from a subalpine Norway spruce [*Picea abies* (L.) Karst.] forest in Switzerland showed growth releases in the edge trees after one-sided tree exposure to edge conditions, with higher radial growth at the stem base relative to breast height position (Bräker and Baumann, 2006). The authors supposed adaptive belowground growth of the edge trees to the new wind exposure. Hence, after a certain degree of competition-release, adapting tree growth would favor allocation belowground as this response can improve wind firmness of trees, i.e., would be beneficial for tree functionality and structural stability.

The effects of management on the relative tree-stem growth are related to tree size: small-sized trees react more strongly to thinning or strip cutting than larger trees (Epp et al., 2005; Vitali et al., 2016). The size of trees or their social position in closed stands results mainly from the lower availability of resources they can acquire (Pretzsch et al., 2018). A study in stands in the Solling Mountain region (Germany) showed dominant spruce trees [stem diameter at breast height (*dbh*) higher than 60 cm] to attend the highest root–stem ratios (Bolte et al., 2004). Tall and dominant spruce trees had not only larger root biomass but also showed higher anchorage than smaller and suppressed trees (Bolkenius, 2003). However, only view quantitative results exist about the time needed for spruce trees to acclimate to reduced canopy density (Urban et al., 1994; Pretzsch et al., 2014). Long adjustment periods would make edge trees vulnerable to ecological risks such as wind or snow break (Wonn and O'Hara, 2001) followed by bark beetle attacks (Jurc et al., 2006). So, the intended effects of silvicultural interventions may be outweighed by unintended side effects. A profound assessment of such effects is particularly important for designing future cutting strategies in the spruce forests of Central Europe, as the ongoing warming combined with the highly uncertain wind regime (Lindner et al., 2014) will challenge our ability to evaluate related ecological risks.

To promote natural regeneration, narrow slit-shaped gaps (20–30 m wide) have been cut since the 1980s in many of the Alpine forests of Switzerland dominated by spruce (Brang, 1998). However, 10–15 years after the intervention, in a significant number of cases, the trees surrounding these regeneration gaps were damaged by storm events or bark beetle outbreaks (Streit et al., 2009). As the intensity of natural disturbances in Alpine forests is expected to increase with climate change (Seidl et al., 2014; Bebi et al., 2017), the area covered by forest edges will also increase. This may particularly affect even-aged, spruce-dominated stands with a closed canopy (Stritih et al., 2021). Therefore, the quantification of timing and magnitude of the structural acclimation of remaining edge trees to gap cutting is of general interest.

Aboveground responses of spruce trees to large strip cuttings were recently quantified in high-elevation stands. After cutting, the radial growth temporally increased by 12–60% only in edge trees growing in north-facing sites, with stronger effects in small trees (20–30 cm *dbh*; Vitali et al., 2016). However, it remained unclear how edge conditions influence root growth. This is of particular interest as belowground allocation may optimize the form of the tree not only to enhance resource uptake but also to increase its ability to withstand the altered wind regime. Therefore, in summer 2017, we cored individual trees again on the same experimental sites (Vitali et al., 2016) but took samples from three compartments: stems, representative root collars, and main roots. Annual increment in roots and stems and their allometric relationships were applied as surrogate variables for tree-growth allocation to aboveground and belowground organs (Nikolova et al., 2011; Pretzsch et al., 2014). Although root–stem ratios are only a coarse indicator of acclimation processes affecting carbon (C) allocation (Poorter et al., 2012; Prescott et al., 2020), increment data provide good estimates of belowground and aboveground biomass growth (Vincent et al., 2009). To this end, the following analyses were done:

(1) Assessment of the long-term allometry for stand-interior trees to test if the relative allocation to roots is site- or tree-size-specific.
(2) Comparison of the short-term allometry change in stand interior vs. edge trees 10 years before and 10 years after the cutting year. The factors driving the change in post-cutting allometry were analyzed by statistical modeling.

## Materials and Methods

### Study Area

The study was conducted in summer 2017 on 74 adult spruce trees, growing at two locations in the Upper Rhine Valley and a third one in the Lower Engadine in the Swiss Alps (Grisons, Switzerland). Our sampling used three permanent plots established in a previous study on radial growth changes in spruce after strip cutting (Vitali et al., 2016). Tree age and forest origin (naturally regenerated or planted forest) are known to influence tree allometry (Huxley and Tessier, 1936; Wang et al., 2008); therefore, we selected the stands Furna, Siat, and Sur En, which represent forests with trees originating from natural regeneration (Table 1). The three stands are composed of 100% spruce, and have been affected by neither management activities nor natural disturbances within the 20 years prior to strip cutting. The time since the year of strip cutting varies from 11 to 13 years. The three sites are located at elevations ranging from 1,500 to 1,680 m a.s.l. on slopes with an inclination between 50 and 70%. Siat has a south-facing aspect with winds predominantly from the north, whereas Furna and Sur En have a north-west aspect with winds prevailing from the south (Table 1).

**Table 1:** Site and stand description.

**Site**	**Furna**	**Siat**	**Sur En**
Latitude	46°54′07″N	46°47′58″N	46°48′43″N
Longitude	9°40′16″E	9°09′40″E	10°22′35″E
Elevation (m a.s.l.)	1,680	1,615	1,500
Aspect	North-west	South	North-west
Slope inclination (%)	59	51	72
Edge orientation	NE	E	NE
Main wind directions^a^	SSW/NNE	SSW/NNW	SSW/NNE
Mean annual wind velocity (m s^−1^)	5.6	3.7	3.0
Stand characteristics			
Stand origin	Natural regeneration	Natural regeneration	Natural regeneration
Management type	Selection cuttings	Selection cuttings	Sporadic
Year of last intervention before strip cutting	1990	1974	1960
Year of the strip cutting	2004	2004	2006
Average strip width (m)	70	70	35
Stand density (n ha^−1^)	332	363	644
Growing stock (m^3^ ha^−1^)	1,029	668	400
Basal area (m^2^ ha^−1^)	71.2	47.6	34.6
Dominant tree height (m)^b^	35.6	33.1	28.4
Stem *dbh* of dominant trees (cm)	61.5 ± 0.5	56.3 ± 0.4	41.2 ± 0.9
Site index^c^	22	20	16
Climate^d^			
Mean annual air temperature (*Ta*, °C)	3.5	3.4	4.2
Min/Max monthly annual air temperature (°C)	−0.2/7.8	0/7.6	−0.5/10.0
Mean air temperature during growing season (°C)	11.1	11.0	12.8
Annual precipitation sum (*Pr*, mm)	1,290	1,496	809
Precipitation sum during growing season (mm)	463	480	310
Annual De Martonne index (DMI)	96	111	57
Soil characteristics^e^			
Soil type	Albic Podzol	Albic Podzol	Skeletic Cambisol
Rooting depth (cm)	80	80	85
Available water capacity (mm/80 cm soil depth)	166	136	82
Humus type	Mor	Mor	Moder

a *Modeled wind data according to Windatlas (2017), maximal wind velocity is derived from the site-specific wind rose (Windatlas Schweiz, 2017)*.

b *Average height of the 100 largest trees per hectare. The tree parameter data are given with the mean ± std. error. Plot size is 0.35 ha (Furna), 0.49 ha (Siat), and 0.45 ha (Sur En) ha; calipering threshold 8 cm*.

c *Site index according to Keller (1978), i.e., dominant tree height at age of 50 years. Low values indicate low site productivity*.

d *Climatic data were averaged over the period 1970–2016. The growing period is defined as June 1 to August 31*.

e *Soil description represents soil characteristics of the stand interior*.

In this study, Furna is the oldest (nearly 270 years old), and Sur En is the youngest (nearly 160 years old) stand. Furna and Siat were traditionally managed by repeated selection cutting, while Sur En was only sporadically managed. With an average width of 35 m, the strip in Sur En was two times smaller than the strips in Furna and Siat (Table 1). The edges of the three strips are oriented to East or North-East and receive direct radiation in the morning and around noon. Winds are prevailing from South-South-West or North-North-East, and Furna is the windiest site with a mean annual wind velocity of 5.6 m s^−1^ (Table 1).

The spruce stands differed in stand characteristics and climate. Furna has the lowest tree density (332 trees ha^−1^, caliper cut-off size 8 cm) but the highest growing stock and basal area among all stands (1,029 and 71.2 m^2^ ha^−1^, respectively; Table 1). Siat has a similar stand density to that in Furna (i.e., 363 trees ha^−1^), but a lower growing stock and basal area (668 and 47.6 m^2^ ha^−1^, respectively), and Sur En have the highest stand density (644 trees ha^−1^) with the smallest growing stock (400 m^3^ ha^−1^) and lowest basal area (34.6 m^2^ ha^−1^). According to the site index of Keller (1978), showing the dominant tree height at an age of 50 years, Furna is the most productive site, and Sur En is the site with the lowest productivity (site index of 22 and 16, respectively; Table 1). Stand data were assessed according to the instructions from the Experimental Forest Management program (Forrester et al., 2019).

**Figure 1:**
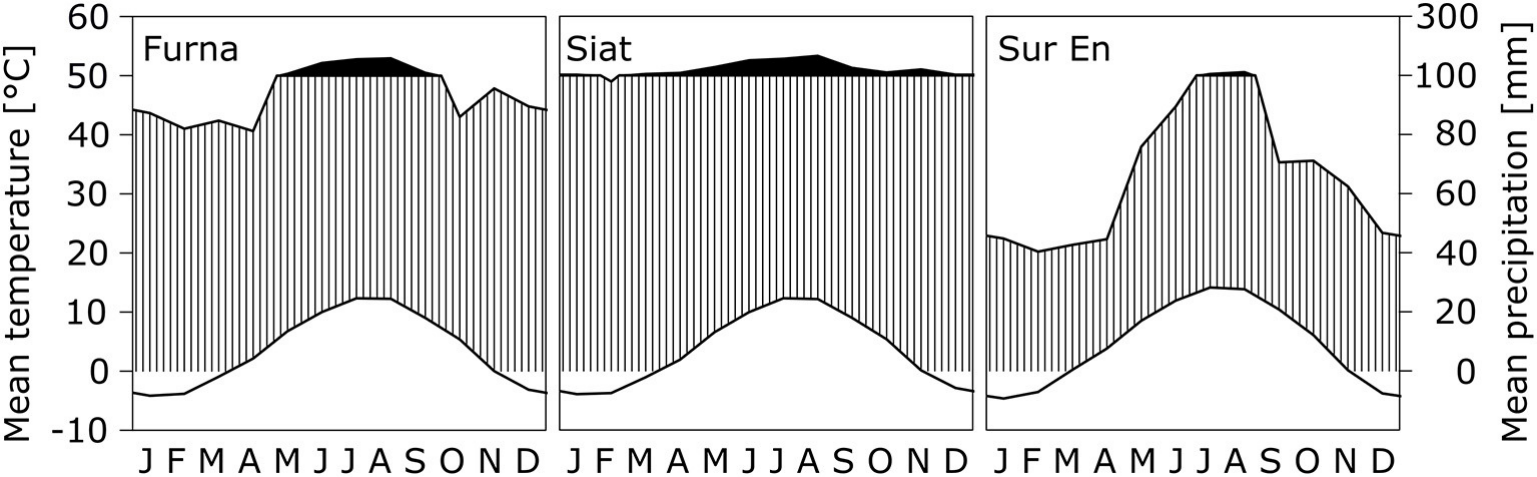
Walter-Lieth climate diagrams representing the long-term averages (from 1970 to 2016) of monthly air temperature *Ta* (upper line) and monthly precipitation sum *Pr* (lower line) at the study sites Furna, Siat (both Upper Rhine Valley), and Sur En (Lower Engadine). The vertical axes for *Ta* and *Pr* are scaled with a ratio of 1:2, as after Walter and Lieth (1960–1967) a month is classified as arid if *Ta* in °C exceeds two times *Pr* in mm. Black areas indicate periods with *Pr* > 100 mm, and white areas show periods with *Ta* < 0°C. Note that the vertical axis is broken at *Pr* = 100 mm.

Daily means of air temperature (*Ta*) and precipitation sum (*Pr*) were obtained for each study site using the DAYMET software (Thornton et al., 1997). Furna and Siat from the Upper Rhine Valley have similar climatic characteristics with an annual mean *Ta* of nearly 3.5°C and an annual *Pr* amount of 1,300–1,500 mm. Sur En is located in the Lower Engadine, which is one of the driest regions of Switzerland and is characterized by a continental climate. To identify the water availability of the study sites, the annual de Martonne aridity index (DMI) was used (Maliva and Missimer, 2012). Furna and Siat are classified as “excessively humid” sites, whereas Sur En with DMI <60 is a “very-humid” site (Table 1; for DMI classification refer to Pellicone et al., 2019). This results from the relatively low amount of *Pr* in the continental Lower Engadine (Figure 1).

### Sampling Design

#### Characteristics of the Sample Trees

Eleven to sixteen vigorous dominant or codominant trees without visible damage by pathogens or rockfall were selected from the edge zone and the stand interior of each study site as sample trees. Trees with crown perimeters exposed to the cutting area were defined as edge trees and the remaining trees as stand-interior trees. We measured tree height *h, dbh*, stem diameter at a position of coring *ds*, live crown ratio (*Lc*) (calculated as the percentage of live crown length to the total tree height), and two perpendicular diameters of the sampled main roots at the coring positions *dr*_1_ (in horizontal direction) and *dr*_2_ (in vertical direction). As a measure of tree stability, we calculated the individual *h* to *dbh* ratio (*h/d* ratio). In addition, for documentation purposes, we sketched the main root architecture of each sampled spruce in relation to the cutting area.

#### Sampling Procedure

Core samples were taken from all trees using 400 mm Haglöf increment borers (5 mm in diameter). Three parts of the trees were sampled: stems, root collars, and main roots. In the stems, the same sampling procedure was applied to edge and stand-interior trees. Each sample tree was cored two times at 1.0 m stem height to avoid damage at the position of future *dbh* measurements on the future permanent plots. The stem increment cores were taken from two opposing directions perpendicular to the slope direction to minimize the potential bias caused by any reaction wood.

A sampling of root collars and roots differed between edge and stand-interior trees. From each edge tree, four horizontal main roots were sampled: two roots growing in the direction of the cutting area and two additional roots in the direction of the stand interior to account for effects of wind load and possible differences in soil water/nutrients availability on cambial activity. From the stand-interior trees, two main roots were sampled: one main root facing toward the cutting area and one growing in the opposite direction to avoid eventual bias from different wind loads due to the nearby edge. Roots that were nearly vertically oriented were not cored.

Each sample root was first cored at the root collar at a maximum distance of 10 cm from the trunk edge. The same root was then excavated, keeping damage to the soil as low as possible, and a second increment core was taken from the top at a distance of 30–100 cm from the trunk edge. In this range, missing rings are comparably rare, and cross-sections exhibit small eccentricity (Gärtner, 2007; Wrońska-Wałach et al., 2016). Horizontal *dr*_1_ and vertical *dr*_2_ root diameters were measured at the coring position by a slide caliper (precision 1 mm) to get an estimate of mean root diameter *dr*.

In the laboratory, cores that could not be measured due to rot were discarded from further analyses (Supplementary Table 2). This procedure provided an average of 3.8 main root cores per edge tree, and 1.9 main root cores for the trees from the stand interior. According to our sketches of the main root architecture, each tree had an average of 5.2 horizontal main roots available for coring. This means that in the present study we have analyzed 40% (for the stand-interior trees) to 75% (for the edge trees) of all horizontal main roots available for coring.

After a windthrow event in Sur En (August 8, 2017), several trees from the study plot and the surrounding forest were uprooted, exposing the root system. We used this chance to obtain an estimate of the main root eccentricity. To this end, 30 disks of eight roots and five trees were cut in distances of 30–160 cm from the trunk edge. The samples were then sanded and growth eccentricity from the pith was measured in four directions (for methodological details see Montagnoli et al., 2020) and related to the distance from the trunk edge. The eccentric growth of the main roots ended more than 100 cm from the trunk edge. At a closer distance, root radii at the upper part of the root were on an average 40% wider than the mean radius of the corresponding cross-sectional root disk (data not shown).

### Tree-Ring Measurement and Dating Procedure

The increment cores were prepared with a core-microtome (Gärtner and Nievergelt, 2010) to maximize annual ring visibility. Ring widths were measured to the nearest of 1/100 mm using the Lintab 5 measuring system in connection with the software TSAP-Win (Rinn, 2012). As color differences between earlywood and latewood were occasionally low in the roots, chalk was applied to identify the annual ring border.

After visual cross-dating, COFECHA (Grissino-Mayer, 2001) was used to statistically check synchronization and dating of each stem series based on site-specific pointer years (Supplementary Table 1). The two stem cores of a tree were averaged to obtain a representative time series of the aboveground increment for each tree. The resulting mean series was used to cross date root collar and root cores of the same tree. For root and root collar cores, high *t*-values and a good optical fit with the stem chronology and the mean site chronologies (per position: edge, stand interior) were used as indicators for dating success. COFECHA routines were not used on the root samples as these often feature too few rings and the results proved to be not helpful for dating (Nikolova et al., 2011). Missing rings were found especially in the root samples, and represented on an average 1.5% of all rings in the specimens. No double rings were recorded across all studied plant compartments.

A total of 71 stems, 209 root collars, and 211 main root ring-width chronologies were built for edge and stand-interior trees on the three sites (Table 2). All cores that did not reach back at least 10 years before the respective year of cutting were excluded from the dataset. In preliminary analyses, no significant difference was found between the post-cutting annual increments related to the direction of root collar or main root growth (data not shown). Therefore, the root collar and main root increment series were averaged to produce one-time series representative for each of the root collars and the main root growth patterns of the respective tree. The common interval of all series was truncated to the period 1970–2016, as this interval enabled us to study the growth reactions of spruce trees before and after the year of strip cutting (i.e., 2004 in Furna nad Siat; 2006 in Sur En; Table 1), and was enough long for cross dating.

**Table 2:** Characteristics of the sampled trees (stems and roots) in summer 2017.

**Tree position**	**Furna**			**Siat**			**Sur En**		
	**Stand interior**	**Edge**		**Stand interior**	**Edge**		**Stand interior**	**Edge**	
*N* sampled trees	15	14		12	11		16	14	
Age of the five oldest trees (years)	269 ± 19	265 ± 7		213 ± 6	204 ± 11		173 ± 3	151 ± 10	
Tree height (*h*) (m)	34.0 ± 1.9	33.4 ± 3.2		30.9 ± 3.3	32.3 ± 4.6		30.0 ± 2.4	25.0 ± 4.2	
Stem diameter (*dbh*) (cm)	57.3 ± 5.5	57.9 ± 9.5		52.9 ± 8.1	57.0 ± 6.8		43.5 ± 6.7	34.8 ± 11.4	
*h*/*d* ratio	59.7 ± 5.2	58.7 ± 7.6		58.9 ± 5.4	56.6 ± 3.8		69.8 ± 7.2	74.9 ± 11.8	
Live crown ratio (*Lc*) (%)	74.7 ± 12.0	74.8 ± 12.5		61.8 ± 12.9	67.2 ± 9.4		62.6 ± 14.1	63.6 ± 10.2	
Root growing direction	Stand	Cut	Stand	Stand	Cut	Stand	Stand	Cut	Stand
Number of sampled roots	26	23	23	21	19	18	28	24	23
Root diameter *dr* (cm)	20.11 ± 9.7	19.2 ± 9.4	16.8 ± 7.6	21.1 ± 9.2	20.5 ± 9.6	19.4 ± 8.2	19.7 ± 8.0	18.0 ± 8.9	18.5 ± 9.9
Age of root samples (years)	75 ± 32	58 ± 28	73 ± 31	104 ± 48	121 ± 59	94 ± 42	96 ± 31	77 ± 29	72 ± 26

*Data are shown as mean ± SE*.

We used mean sensitivities (MSs; Fritts, 1976) of stems, root collars, and main root growths as indicators for high-frequency growth patterns of each of the studied tree parts to changes in environmental conditions. We calculated MS for each chronology as the difference between two successive rings divided by their mean (Biondi and Qeadan, 2008) using the function *sens1* of the R package “dplR” (Bunn, 2008). Then, the MS was averaged for the period of 46 years (1970–2016) within each studied tree compartment and study site. As a measure of the series intercorrelation, the cross date index (CDI) was calculated for the same period of 46 years using TSAP-WIN. In this study, we show MS and CDI analyses only for stand-interior trees, as the growth patterns of edge trees during the last 10–12 years were additionally influenced by strip cutting.

The ring-width series of stems, root collars, and roots were indexed to remove biological trends (e.g., the age trend) following standard procedures (Cook and Kairiukstis, 1990; Esper and Gärtner, 2001). The indexed chronologies were then averaged per tree compartment, tree position, and site.

### Allometric Analysis

For the allometric analyses, we focused only on stems and main roots as indicators for above- and belowground tree growth (Cherubini et al., 2021). At the root collar, effects of tree swaying and reaction wood as a result of altered wind exposure can be expected (Wrońska-Wałach et al., 2016).

We applied the basic allometric equation (Huxley and Tessier, 1936), which describes how the studied plant organs main root and stem change with plant size:

(1) $$\begin{array}{l} \ln\left(dr_{i}\right)=\alpha_{0}+\;\alpha \;\times \;\ln\left(ds_{i}\right) \end{array}$$

where α_0_ is the allometric factor and α the allometric exponent, and *dr*_*i*_ and *ds*_*i*_ are the root and stem radii for the year *i* from the study period 1970–2016. Stem and root radii were calculated backward by subtracting the measured annual radial increment from the radius at survey time.

Logarithmic transformation of the observed radial increment data reduces the effect of outliers on the results, and the transformed data better meet the statistical assumptions of normal distribution and homoscedasticity (Poorter and Sack, 2012). The biologically relevant term in Equation (1) is the exponent α, which covers both radial increment and plant proportions in the long term, and equals 1.0 when plant growth is in a steady-state (Poorter et al., 2012). In this study, we only used the trees from the stand interior, representing undisturbed long-term growth. As this study is not based on biomass assessment and because of the eccentric form of the roots, α overestimates the relative increase of root biomass growth. Therefore, in the present work, α was used as a surrogate variable to analyze long-term changes in tree allometry between stems and roots, and not as a predictor for the absolute biomass allocation to these plant organs (Nikolova et al., 2011).

We applied the value pairs *dr*_*i*_, *dr*_*i*−1_, and *ds*_*i*_, *ds*_*i*−1_ from consecutive years to calculate the period-wise slope α′_*i*_, which represents the short-term allometry change between root and stem of individual trees within 1 year:

(2) $$\begin{array}{l} {\alpha^{\prime }}_{i}=\frac{\ln\left(\frac{dr_{i}}{dr_{i-1}}\right)}{\ln\left(\frac{ds_{i}}{ds_{i-1}}\right)} \end{array}$$

Both radii *dr* and *ds* for the year *i* were calculated from raw non-detrended ring widths.

Furthermore, we quantified the allometric changes induced by strip cutting in terms of percentage change in allometric slope α′_*i*_ (PCA, %), calculated as the relation between α'_*i*_ prior to (*A*_*p*_) and after (*A*_*a*_) the year of cutting. To this end, we used the formula:

(3) $$\begin{array}{l} PCA=\frac{A_{a}-A_{p}}{A_{p}}\;\times 100 \end{array}$$

where *A*_*p*_ is the mean α′_*i*_ during the 10 years before the cutting, and *A*_*a*_ that during the 10 years after the cutting. Positive PCA-values indicate enhanced allocation to roots instead of stems following strip cutting. In this way, we have broadened the analysis of Vitali et al. (2016), who quantified the growth release of spruce trees from the same study sites as the percentage change in the mean annual radial increment between the two intervals, based on information from stems only.

### Statistical Analyses

We took the nested structure of our data (on a tree level) into account and applied linear mixed models to compute the allometric exponent α for each study site. To this end, we tested for a difference in the root–stem allometries of spruce trees growing in the stand interior:

(4) $$\begin{array}{l} \ln\left(dr\right)=\;\alpha_{0}+\;\alpha_{1}\times \ln\left(ds\right)+\alpha_{2}\times \text{site}+\alpha_{\text{3}}\times \text{ln}\left({\text{ds}}_{\text{j}}\right)\\ \;\times \text{site}+b \end{array}$$

where *dr* and *ds* are the roots and stems radii, α_0_ (allometric factor), α_1−3_ represent the fixed effects (i.e., the allometric coefficients; Equation 1), and *b* considers the random effects on the tree level. In this model, we included the interaction of “stem radius” × “site” to test if the long-term allometry of stand-interior trees is site-specific. Linear mixed models were fitted with the *lme4r* function from the “lme4” package in R. For multiple pairwise comparisons (contrasts), the *HSD.test* function of the “agricolae” package in R (Mendiburu, 2020) was applied.

To explain the change in PCA on a tree-level after strip cutting, we applied linear models with a backward step-wise selection. In the initial models, “stem radius prior to cutting” (*Rp*), “tree position,” and “site” were included as fixed factors. We included *Rp* to test whether growth allocation following cutting depends on tree size prior to cutting.

(5) $$\begin{array}{l} PCA=c_{0}+\;c_{1}\times Rp+c_{2}\times \text{tree position}+c_{3}\times \text{site}+c_{4}\\ \;\times \text{tree position}\times Rp+c_{5}\times \text{tree position}\times \text{site} \end{array}$$

where *Rp* is the stem radius prior to cutting, *c*_0_ is the intercept, *c*_1−5_ represents the fixed-effect coefficients. The interactions “tree position” × “*Rp”* and “tree position” × “site” were tested as we were interested to understand if the cutting effects on trees growing at different positions are size- or site-specific. Outliers with Cook's distance > 8/*N* (*N* = dimension of the dataset) were excluded from the analysis and the model was then refitted (Crawley, 2012). Outlier exclusion (one edge tree in Siat and one stand-interior tree in Sur En) did not change the model outcome but improved the model fit. Model selection was done by the function *stepAIC* of the R package “MASS” (Ripley, 2021), and the model with the lowest Akaike Information Criterion (AIC) was retained as a final model. The interaction plot was visualized by the function *interact_plot* of the R package “interactions” (Long, 2021).

Parameter coefficients and fit (*R*^2^) were used to evaluate model effects and suitability. Wilcoxon rank-sum test was applied to test differences in PCA of stand-interior and edge trees within each site after strip cutting. This and all other computations were performed by R version 3.4.4 (R Development Core Team, 2018).

## Results

### Characteristics of the Sampled Trees

The oldest trees were found in Furna (up to 270 years), whereas trees in Sur En were the youngest, with a maximum age of about 160 years (Table 2). The largest sample trees were recorded in Furna (mean values of about 34 m in height and 58 cm in *dbh*), the trees in Siat were only slightly smaller, and the smallest trees were found in Sur En (height: 25–30 m, dbh: 35–44 cm), with somewhat larger dimensions in the stand interior than at the edge. The resulting mean *h/d* ratios were <60 in Furna and Siat, but in Sur En, mean *h/d* ratios were 70 and 75 for stand-interior and edge trees, respectively (Table 2). The sampled spruces had a *Lc* of 62–75% with maximum values in Furna. On all sites, *Lc* was similar for edge and stand-interior trees (Table 2).

The average diameter of the main roots sampled varied largely within stands, tree positions, and growing directions and ranged between 16.8 and 21.1 cm while not showing any significant patterns related to their growing direction. Main roots were on an average 58–121 years old at the position of coring with the oldest roots detected in edge trees in Siat. Interestingly, the youngest roots were sampled in the oldest and coldest stand Furna, but had similar dimensions (diameters) as the roots sampled from the other two sites (Table 2).

### Long-Term Growth

#### Stem, Root Collar, and Root Growth

In trees from the stand interior, the mean annual tree-ring width of stems was higher in Furna (1.2 mm) and Siat (1.3 mm) than in Sur En (0.9 mm) (Supplementary Table 3; Supplementary Figures 1–3). Annual tree rings were widest in the root collars (from 1.8 cm in Sur En to 2.7 cm in Siat) and narrowest in the main roots (from 0.7 mm in Sur En to 1.0 mm in Siat; Supplementary Figures 1–3).

The MS of stand-interior trees was lowest in stems (0.14–0.23), followed by root collars (0.19–0.27), and roots (0.27–0.36; Supplementary Table 3). Overall, MS was highest in the driest site Sur En and lowest in Furna. Series intercorrelation captured by CDI was highest in stems (0.67–0.79) and lowest in roots (0.46–0.55) in all study sites. The highest MS and CDI values were found in all studied tree compartments of trees from Sur En.

#### Root–Shoot Allometry of Stand-Interior Trees

**Figure 2:**
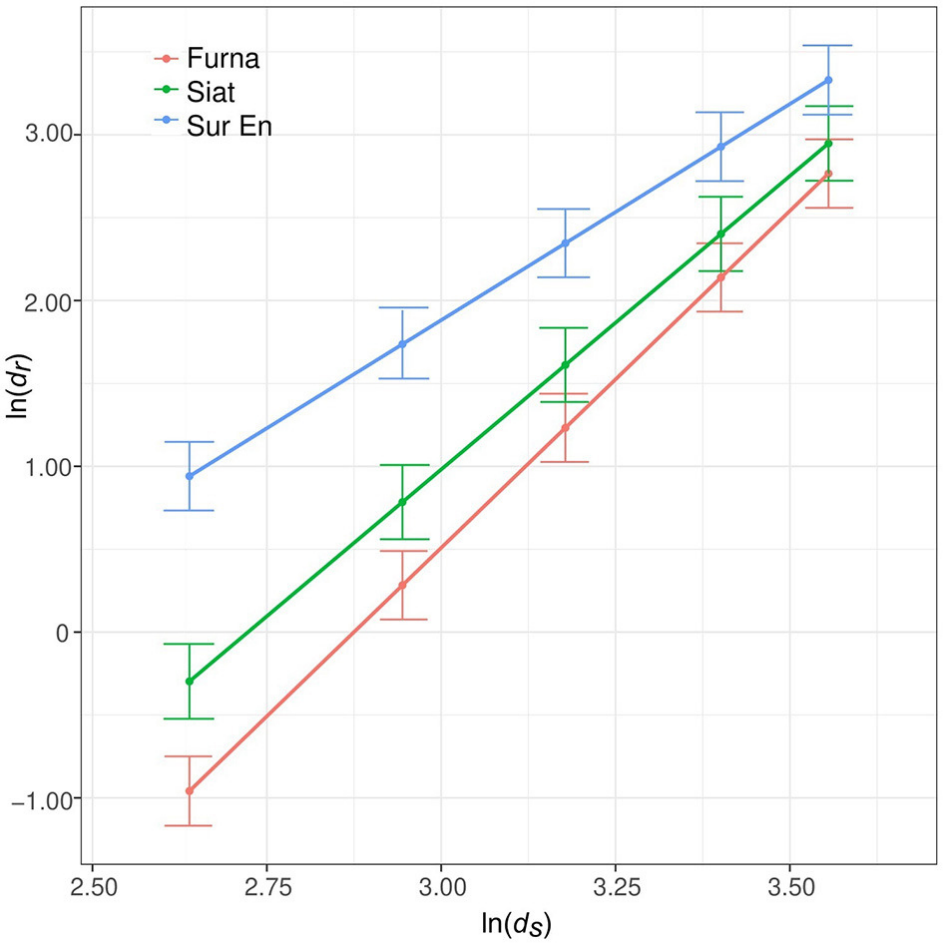
Root–stem long-term allometry α (1970–2016) from the fitted model according to Equation (4). Its coefficients are shown in Table 3. Points indicate model estimates at five different stem radii covering a range of *ds* from 13 to 37 cm, and whiskers are the SE, with *dr* and *ds* being the root and stem radii (mm).

The long-term tree allometry described by the allometric exponent α differed between sites and was highest in Furna and lowest in Sur En (Figure 2, Table 3). Its values between 2.60 (in Sur En) and 4.06 (in Furna) illustrate a long-term site-dependent increase of growth allocation to roots with increasing stem diameter.

**Table 3:** Estimated fixed effects for the long-term allometry (α) model (1970–2016) with the response variable ln(*dr*) of the stand interior trees (*n* = 37) in relation to ln(*ds*) and site [Furna, Siat, and Sur En; Equation (4)], with *dr* and *ds* being the root and stem radii (mm).

	**Estimate**	**Std. error**	***t*-Values**
(Intercept)	−11.68	0.29	−40.83^***^
ln(*ds*)	4.06^a^	0.06	66.31^***^
Siat	2.04	2.04	4.99^***^
Sur En	5.74	0.40	14.30^***^
ln(*ds*) × Siat	−0.52^b^	0.09	−6.10^***^
ln(*ds*) × Sur En	−1.46^c^	0.09	−16.37^***^

a,b,c *indicate significant differences between sites (compared to Furna)*.

*Significance level*

*** *p < 0.001*.

### Effects of Strip Cutting on Tree Growth

#### Radial Growth of Edge and Stand-Interior Trees Before and After Strip Cutting

In Furna, the raw-data mean chronologies (Supplementary Figure 1) of edge trees show an overall increase of average annual ring width after strip cutting with the highest effects in root collars and roots. In Siat, no change in growth was detected in any of the studied tree compartments (Supplementary Figure 2), whereas in Sur En, all tree compartments reacted clearly with an increased average post-cutting radial increment (Supplementary Figure 3).

**Figure 3:**
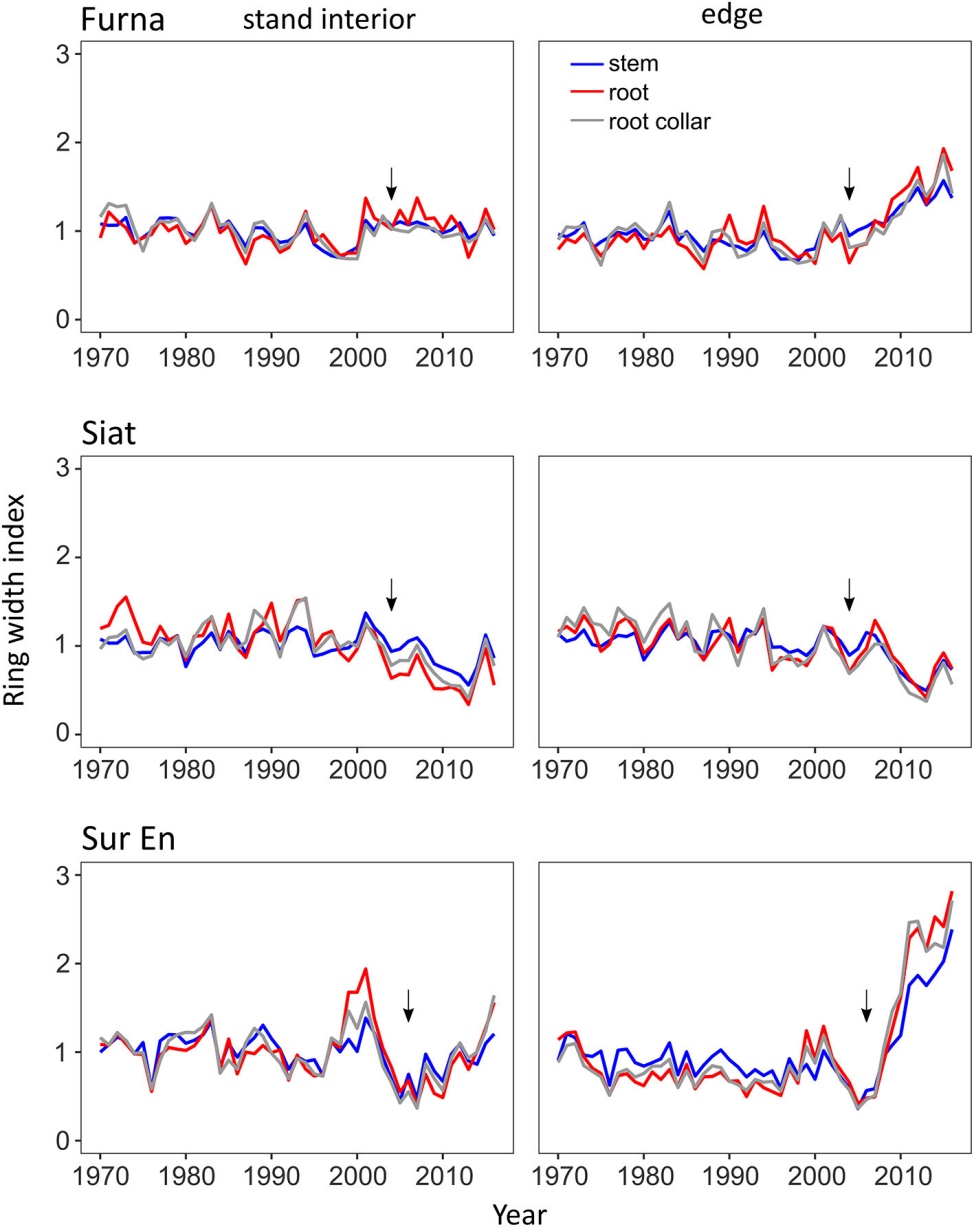
Mean chronologies of the ring-width index for stand interior and edge trees in the three study sites Furna, Siat, and Sur En for the period 1970–2016. Arrows indicate the cutting year.

Before strip cutting, the year-to-year variation and the overall trend of indexed ring widths were similar in stand-interior and edge trees at all study stands and in all tree compartments (Figure 3). The distinct differences in the growth pattern of stand-interior and edge trees began to appear 2–3 years following the strip cutting (in Furna and Sur En) and lasted until the last measured year 2016. In Siat, trees from the stand interior and the edge showed similar growth patterns, which seemed independent of cutting.

#### Change in the Root–Shoot Allometry After Strip Cutting

**Figure 4:**
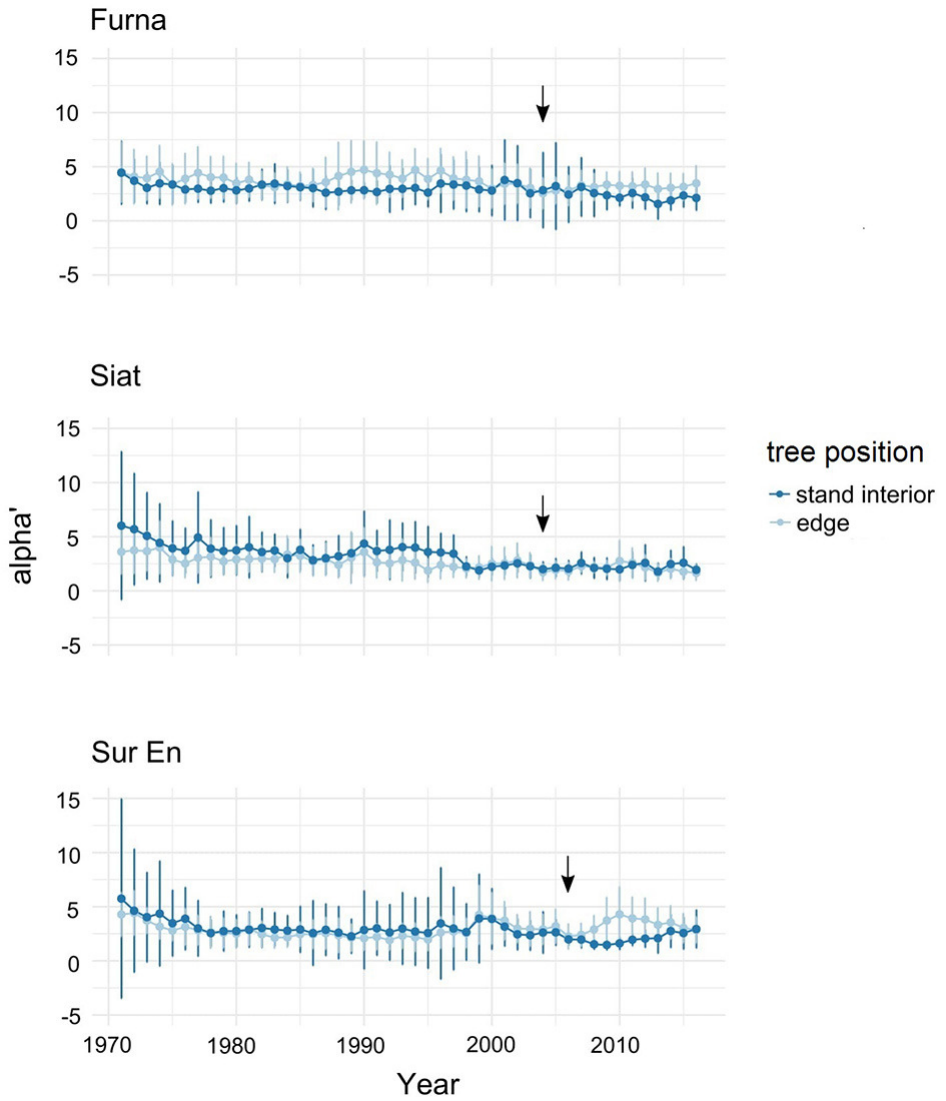
Annual allometric slope α' (alpha' ± standard deviation) in edge and stand interior trees at the study sites Furna, Siat, and Sur En. Alpha' is calculated according to Equation (2) for the period 1971–2016. Arrows indicate the year of strip cutting.

Before cutting, the short-term allometry α' showed temporary differences for edge and stand-interior trees in Furna (in the period 1985–1995) and Siat (in the periods 1970–1980 and 1990–1997) with a high variance among both tree groups (Figure 4). A distinct increase in α' after strip cutting was detected in the edge trees of Sur En, and only a small change occurred in Furna, which indicates a higher allocation of growth to the roots than to stems on both sites. This change in growth started 2 years after cutting but disappeared after 7 years.

**Figure 5:**
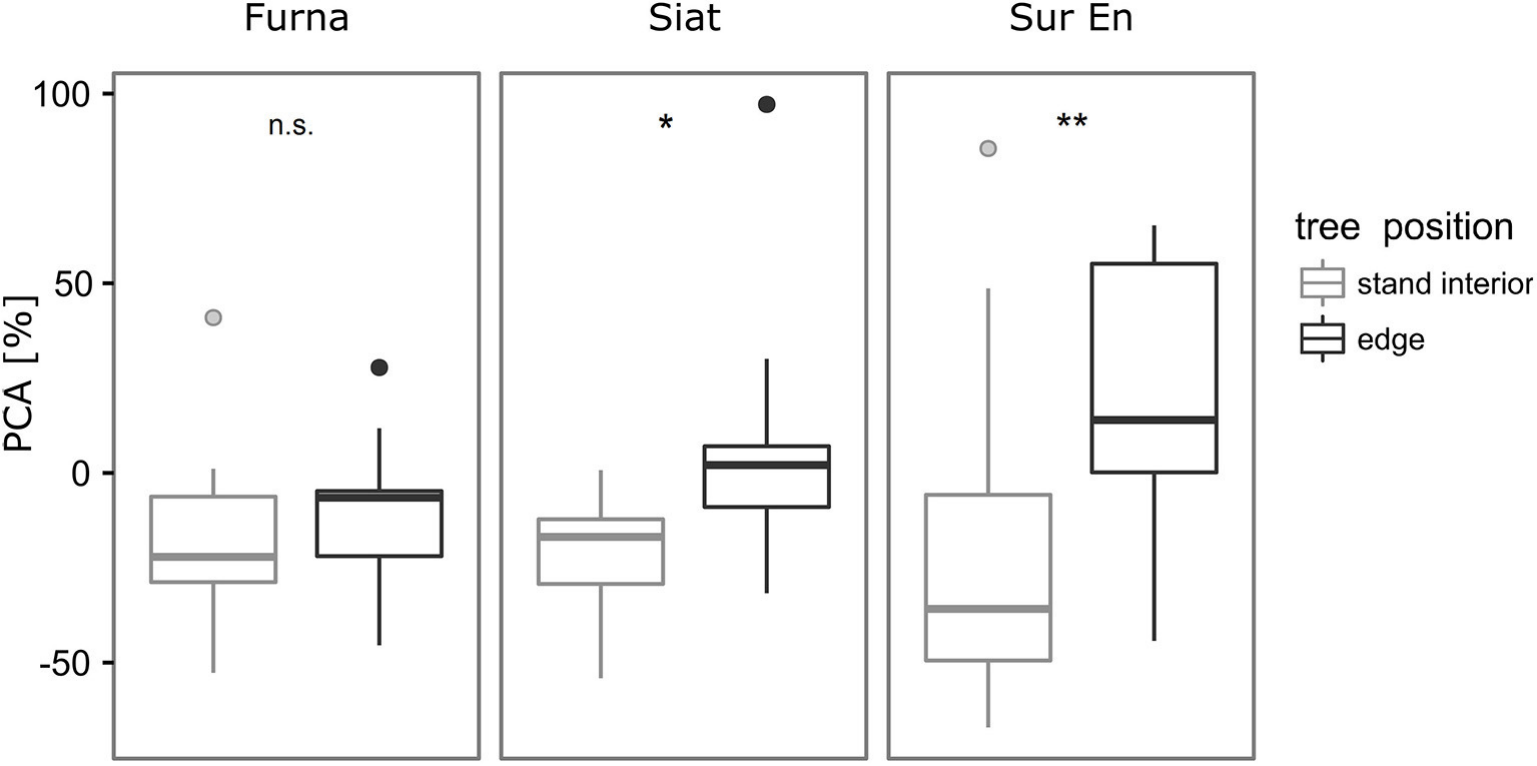
Box plots of the percentage change in allometric slope α'_*i*_ (PCA, %) of stand interior and edge trees after strip cutting. Wilcoxon rank-sum test was applied to test differences in PCA of the stand interior and edge trees within each site, significance levels as follows: *p ≤ 0.05, **p < 0.01, n.s. (not significant, *p* > 0.10). Individual points represent outliers.

No change in PCA was detected in Furna, while significantly more belowground growth following cutting was found in edge trees from Siat and Sur En (Figure 5). Variation of PCA was strongest in Sur En in both stand-interior and edge trees. PCA was negative in the stand-interior trees from all sites, and positive in edge trees only from Siat and Sur En.

#### Factors Affecting the Changes in the Short-Term Allometry

**Figure 6:**
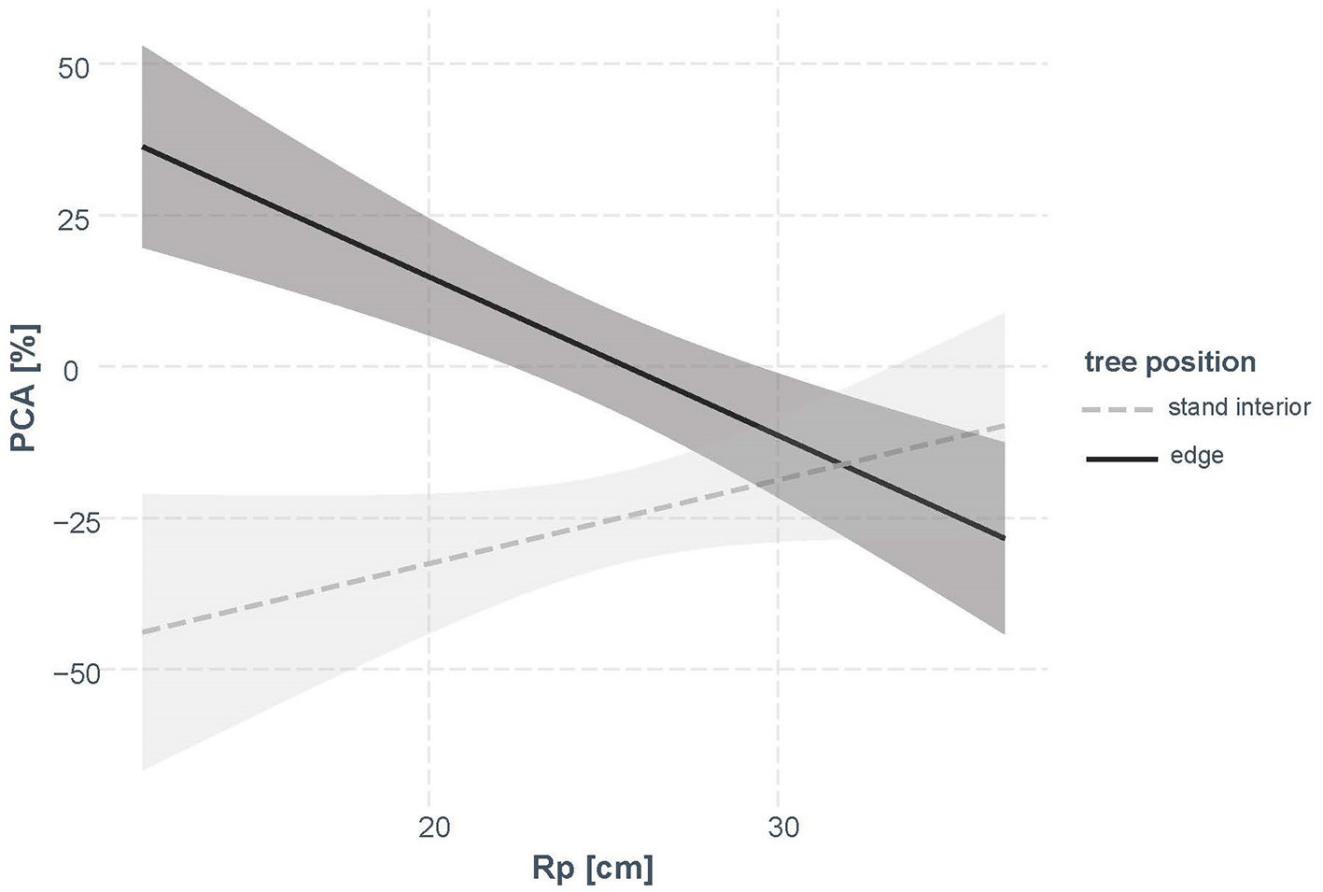
Interaction plot of “tree position” and *Rp* (stem radius prior to cutting) according to the statistical model (Table 4). CI (95%) are shown in gray.

In contrast to as could be expected from Figure 5, the factor “site” not significantly related to the change in PCA in spruce and was therefore omitted during model selection. Our final model was based on the variables “tree position,” *Rp*, and included the interaction term “tree position” × *Rp*. The model explained 46% of the variation in PCA (Table 4). Nearly 25% of the variation was due to “tree position” and the other 20% were explained by the interaction of “tree position” with *Rp*. Allocation to roots generally decreased after the year of cutting in the stand-interior trees, but increased in the edge trees, with stronger allocation to main roots in small edge trees (Figure 6).

**Table 4:** Estimated fixed effects of the model for predicting the percentage change in period-wise allometric slope α′_*i*_ (PCA).

	**Estimate**	**Std. error**	***t*-Value**
(Intercept)	−60.15	20.31	−2.96^**^
Tree position “edge”^a^	127.38	24.98	5.10^***^
*Rp*	1.38	0.78	1.77^*^
Tree position “edge” × *Rp*	−4.00	0.96	−4.15^***^

*Only significant effects are shown R^2^ = 0.46. The variable selection and the variables are described in section Statistical Analyses*.

*Significance levels*:

* *p ≤ 0.05*,

** *p < 0.01*;

*** *p < 0.001*.

a *Compared to tree position “stand interior”*.

## Discussion

Strip cuttings are increasingly being practiced in the Swiss Alps to initiate natural regeneration in high-elevation spruce-dominated stands (Streit et al., 2009). The success of this approach is closely linked to the acclimation reaction of the remaining trees growing along the created forest edges, as post-cutting destabilization of the stand may occur (Nielsen, 1995; Girona et al., 2019). Therefore, the present study focuses on the post-cutting growth reaction of stems, root collars, and roots in edge spruce trees in relation to site and tree characteristics. Growth adjustments can optimize tree form for better resource uptake, but may simultaneously be inadequate to enable newly exposed trees to withstand stronger wind forces (Hanewinkel et al., 2015). In the following sections, we discuss the general growth patterns of spruce in the three studied tree compartments (stem, root collar, and roots), the long-term whole-tree (i.e., root–stem) allometry as depending on site and tree characteristics, as well as the factors controlling the allometric post-cutting (i.e., short-term) responses of edge trees. The possible site-and stand-specific effects of strip cutting on the tree and stand stability in high-elevation spruce forests are summarized as implications for practical applications.

### Growth Patterns of Spruce Trees

In this study, we compare increments of stand-interior spruce trees from three Alpine stands differing in site characteristics and productivity. In cold climates such as those of the high-elevation forests, primarily temperature has been shown to control and stimulate tree radial growth (Hartl-Meier et al., 2014). However, the most narrow tree rings with the highest variability of annual growth were found at the comparatively warm but dry and least productive site Sur En. Thus, the high growth sensitivity to site fertility and water supply, which is typical for spruce from lowland forests (Nikolova et al., 2011; Van der Maaten-Theunissen et al., 2013) seems to be valid also in specific high-elevation locations.

The widest annual increments were detected in the root collars, which is the most important component in stabilizing the tree and conferring its resistance to uprooting (Nielsen, 1995; Stokes, 2002). A high increment in the root-collar zone resulting in higher biomass accumulation is most likely caused by increasing wind sway of the aboveground part when trees become dominant and more exposed to wind stresses (Ennos, 1993). The most uniform course in individual root-collar growth in the stand-interior trees at Sur En may reflect, alongside the less variable individual biomass productivity under continental climate conditions, the lowest wind firmness of the spruce trees within this stand. This low variation seems to reflect a failure to achieve individual adaptation to wind forces on this site and may relate to the higher stem density and structural homogeneity in the stand in Sur En as compared to the intensively thinned sites, Siat and Furna. In addition, openings in closed spruce stand relying on collective (i.e. tree-group) stability from the neighborhood may make trees after sudden exposure to edges more susceptible to subsequent disturbances (Nielsen, 1995). This destabilization due to management interventions might have long-term effects on forest resistance if adjustments in edge trees need long time, and disturbance frequency, e.g., due to climate change, simultaneously increases as predicted for North European forests (Olofsson and Blennow, 2005; Blennow and Olofsson, 2008).

### Long-Term Allometry in Different Site Conditions

In this study, the allometric exponent α, which represents the long-term root–stem allometry at the tree level, was site-specific, with the highest allocation to roots in Furna (4.06) and the lowest in Sur En (2.60). The long-term allometry of stand-interior trees likely follows the increase of site productivity, with biomass allocation to roots increasing with *dbh* (i.e., tree size). These results support the allometric biomass partitioning theory (Enquist and Niklas, 2001, 2002), which postulates resource allocation patterns between different organs to differ with plant size. Furthermore, Furna is the windiest among the sites studied. The outstanding large trees that are always exposed to the wind might have additionally increased the relative allocation to roots permanently adjusting to physiological, i.e., enhanced transpiration demand, and biomechanical (i.e. dynamic bending) stresses (Telewski, 2006).

In other studies, much lower α-values of 0.3–1.24 were found in woody and herbaceous plants (Müller et al., 2000; Enquist and Niklas, 2002; Mokany et al., 2006). The higher α-values from this study indicate much higher allocation to roots. Part of this may be attributable to an overestimation due to the eccentric growth of the sampled roots, their upper radii being on an average 40% wider than the mean radius of the corresponding cross-sectional root disc (cf. 48% for lowland spruce trees; Nikolova et al., 2011). Even after correction of α with the overestimation factor of 1.90, the long-term α estimates from this study (i.e., 2.14 for Furna, 1.86 for Siat and 1.37 for Sur En) are still higher than those reported for lowland spruce trees on relatively flat terrain (1.0–1.3; Nikolova et al., 2011). However, the values found here fit well to biomass estimation models parametrized for high-elevation spruce forest stands in Central Europe (2.31; Konôpka et al., 2011) or spruce plantations from the Northern Harz mountains (2.36; Drexhage and Gruber, 1999). The high α-values reported from mountain spruce forests might be related to the larger belowground investment due to genotypic adaptation to colder habitats (Oleksyn et al., 1998; Poorter et al., 2012) or for anchorage to stabilize trees on steep terrain (Chiatante et al., 2002; Dumroese et al., 2019) and suggest allometric trajectories to relate to elevation and/or relief. The latter should be proven by whole-biomass assessments *in situ* (cf. Bolte et al., 2004; Wang et al., 2008) and accounted for in-biomass estimation models.

### Post-cutting Allometry Changes

Before strip cutting, short-term allometry α' values transiently differed between edge and stand- interior trees in Furna and Siat only, possibly reflecting post-cutting phases of allometric adjustment in the managed stands (Pretzsch et al., 2014). In Furna, a periodic but minor increase of allocation to roots was detected in the group of edge trees, whereas in Siat, stand-interior trees have been experienced repeated released events in the past. Interestingly, since 1998, the allometry is flattening in Siat with only small individual variability, with the stem showing the highest increments among the studied tree compartments. This indicates that the sampled trees in the south-facing and selectively thinned site Siat had sufficient resources for equalized/balanced growth even before strip cutting. In Sur En, both tree groups had almost the same long-term allometry confirming the absence of management interventions and other significant disturbances after 1960 on this site.

Strip cutting causes abrupt ecological changes along the created forest edges. This kind of silviculture shapes structural contrasts of high magnitude between stocked and unstocked (i.e., cut) areas, thereby increasing disturbance severity by more overstory mortality (e.g., Goode et al., 2020). In previously thinned stands such as Furna and Siat, the effects on edge tree growth seem to be only moderate, as the edge-to-interior ecological gradient is relatively small (Matlack, 1993). The edge trees in Sur En seem to experience the most abrupt change in growing conditions as indicated by the change in post-cutting root–stem allometry (PCA). In this study, however, statistical analyses (Table 4) yielded no significant site effect on the change in PCA, indicating a general response of the edge trees. In addition, the strongest effects of enhanced post-cutting allocation to roots were detected in small trees. According to the resource limitation theory (Bloom et al., 1985), biomass shifts into roots would indicate primarily improved water/and nutrient availability for the small suppressed trees along edges. Small trees likely profited more than taller trees from the warmer and wetter soils along edges, a root-growth stimulating environment. During the first years after cutting, edge trees with their relatively well-developed crowns (*Lc* 63–75%) might have experienced a surplus of assimilates as a result of rapidly enhanced C uptake under still limited nutrients (e.g., P, N) availability, which excess of photosynthates might have first stimulated fine-root growth and supporting symbiotic associations (Nikolova et al., 2020; Prescott et al., 2020). Over time, such relocation to roots resulted in greater root–shoot ratios (Prescott et al., 2020). In this study, edge-tree adjustment to new light conditions, i.e., the increase of N and P uptake due to enhanced fine-root growth, seems to be completed 7 years after cutting, with stronger effects in formerly suppressed (and more nutrient-limited) small-sized trees.

### Conclusion and Applications for the Praxis

The results suggest long-term allocation to roots to increase with tree size. This outcome supports previous works (e.g., Urban et al., 1994; Stokes, 2002) highlighting the increased allocation of resources to roots as a general mechanism of *Picea* to increase wind firmness with tree size. After one-sided removal of the canopy, small-sized spruces growing as subdominants showed a strongest change in allometry enhancing root growth. A time span of 7–8 years was necessary to allow tress to acclimate to the new growing conditions. Ecologically, the edge influence on tree growth is less important in forests that have been regularly managed and are therefore more structurally heterogeneous (Harper et al., 2005). In the case of homogenous stands with suppressed spruce trees, the post-cutting period is characterized by the high vulnerability of small-sized edge trees to windthrow and/or snow-/ice-break (Klädtke, 1999; Bachofen and Zingg, 2001). An increasing post-cutting vulnerability to a disturbance within the period of acclimation, e.g., by more overstory damage or mortality of subdominant edge trees, may represent a risk for stand integrity, as secondary risk factors such as bark beetle attacks might be provoked. In times of climate change with a predicted increase of extreme events like drought or biotic diseases, strip-cuttings in dense, unmanaged spruce forests should therefore be executed after a careful examination of associated risks.

The results from our case study contribute to a better understanding of the structural acclimation of spruce trees from high-elevation forests to new forest edges. However, for a more mechanistic understanding, an analysis of the interplay of environmental drivers such as light or water availability on the physiological responses of edge trees using tree-ring stable isotopes (Cherubini et al., 2021) would help.

## Data Availability Statement

The original contributions presented in the study are included in the article/Supplementary Material, further inquiries can be directed to the corresponding author/s.

## Author Contributions

PN initiated and designed the study, provided project administration and supervision, and wrote the draft version of the manuscript. JG was the main responsible for lab and fieldwork. PN and JG analyzed the data. PN, PB, HG, SZ, and PC provided interpretations. PN, HG, PB, and SZ contributed substantially to the funding success. All authors contributed to the writing of the manuscript.

## Conflict of Interest

The authors declare that the research was conducted in the absence of any commercial or financial relationships that could be construed as a potential conflict of interest.

## Publisher's Note

All claims expressed in this article are solely those of the authors and do not necessarily represent those of their affiliated organizations, or those of the publisher, the editors and the reviewers. Any product that may be evaluated in this article, or claim that may be made by its manufacturer, is not guaranteed or endorsed by the publisher.
