# Supplementary material for: Changes in Root–Shoot Allometric Relations in Alpine Norway Spruce Trees After Strip Cutting
Source: Front Plant Sci. 2021 Aug 27;12:703674. doi: 10.3389/fpls.2021.703674 (PMC8429809; doi:10.3389/fpls.2021.703674)

Supplementary Material

1 Supplementary Data

2 Supplementary Figures and Tables

Supplementary Table 1. Pointer years were optically defined and used for crossdating the ring width series. Negative pointer years are red, positive years are blue.

Year/ Site	1970					1975					1980					1985					1990					1995					2000					2005					2010					2015	
Furna														blue			red								blue							blue															
Siat											red											red				red																					
Sur En							red								red																					red											

Supplementary Table 2. Number of analysed cores per site, tree position and tree compartment.

Site	Tree position	Tree compartment	Number of analysed cores[1]	Number of omitted cores[2]
Furna	Edge	Stem	22	6
		Root collar	48	8
		Root	46	10
	Stand interior	Stem	26	4
		Root collar	26	4
		Root	26	4
Siat	Edge	Stem	20	2
		Root collar	38	4
		Root	37	5
	Stand interior	Stem	22	2
		Root collar	21	3
		Root	21	3
Sur En	Edge	Stem	26	2
		Root collar	50	6
		Root	55	1
	Stand interior[3]	Stem	26	6
		Root collar	26	6
		Root	26	6

[1] Two individual stem cores build one averaged stem sample.
[2] Increment cores were omitted when not reliably measurable or too short (< 10 years measured before the year cutting).
[3] Two sample trees were lost in Sur En due to the wind-throw event (August 8, 2017).

Supplementary Table 3. Statistical parameters of the stem, root collar and root chronologies in the stand interior trees for the period 1970-2016. Data are shown as mean ± standard deviation, n.d. = not defined.

Site	Tree compartment	Number of analysed cores	Annual tree ring width [mm]	Mean sensitivity	Cross-date index
Furna	Stem	26	1.2 ± 0.2	0.14 ± 0.02	0.68 ± 0.10
	Root collar	26	2.3 ± 0.7	0.19 ± 0.03	0.57 ± 0.18
	Root	26	0.9 ± 0.4	0.27 ± 0.07	0.51 ± 0.14
Siat	Stem	22	1.3 ± 0.3	0.16 ± 0.02	0.67 ± 0.12
	Root collar	21	2.7 ± 1.0	0.24 ± 0.04	0.60 ± 0.13
	Root	21	1.0 ± 0.6	0.36 ± 0.09	0.46 ± 0.24
Sur En	Stem	26	0.9 ± 0.2	0.23 ± 0.05	0.79 ± 0.09
	Root collar	26	1.8 ± 0.7	0.27 ± 0.06	0.75 ± 0.13
	Root	26	0.7 ± 0.3	0.35 ± 0.10	0.55 ± 0.23

2.1 Supplementary Figures

Supplementary Figure 1. Raw ring width series for stem (A), root (B) and root collar (C) in Furna in stand interior and edge trees included in the allometric analyses. The number of analyzed series is shown on Table A3. The resulting mean chronologies are drown in red. The arrow indicates the date of strip cutting.

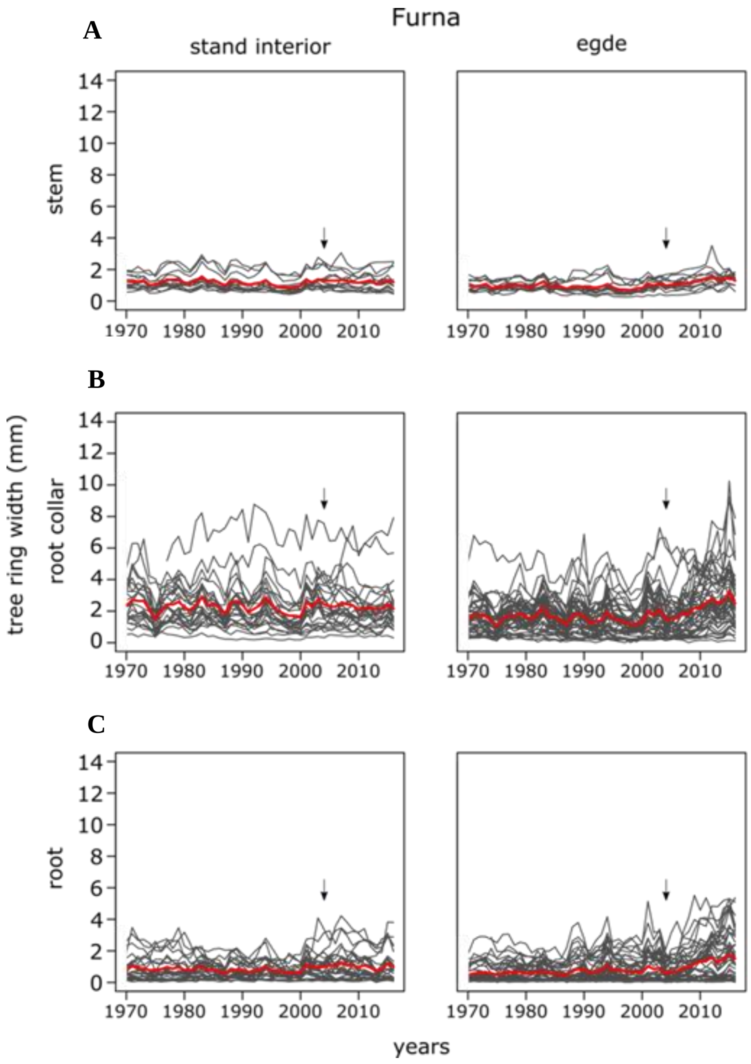

Supplementary Figure 2. Raw ring width series for stem (A), root (B) and root collar (C) in Siat in stand interior and edge trees included in the allometric analyses. Number of analyzed series is shown on Table A3. Resulting mean chronologies are drown in red. The arrow indicates the date of strip cutting.

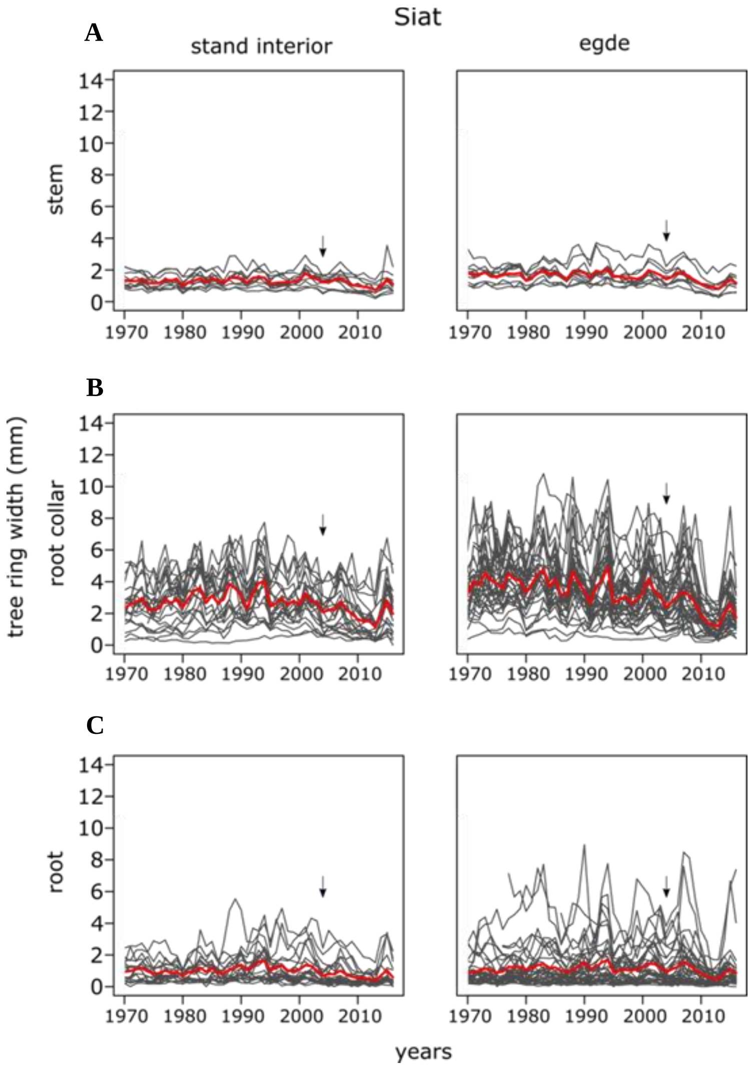

Supplementary Figure 3. Raw ring width series for stem (A), root (B) and root collar (C) in Sur En in stand interior and edge trees included in the allometric analyses. Number of analyzed series is shown on **Table S3**. Resulting mean chronologies are drown in red. The arrow indicates the date of strip cutting.

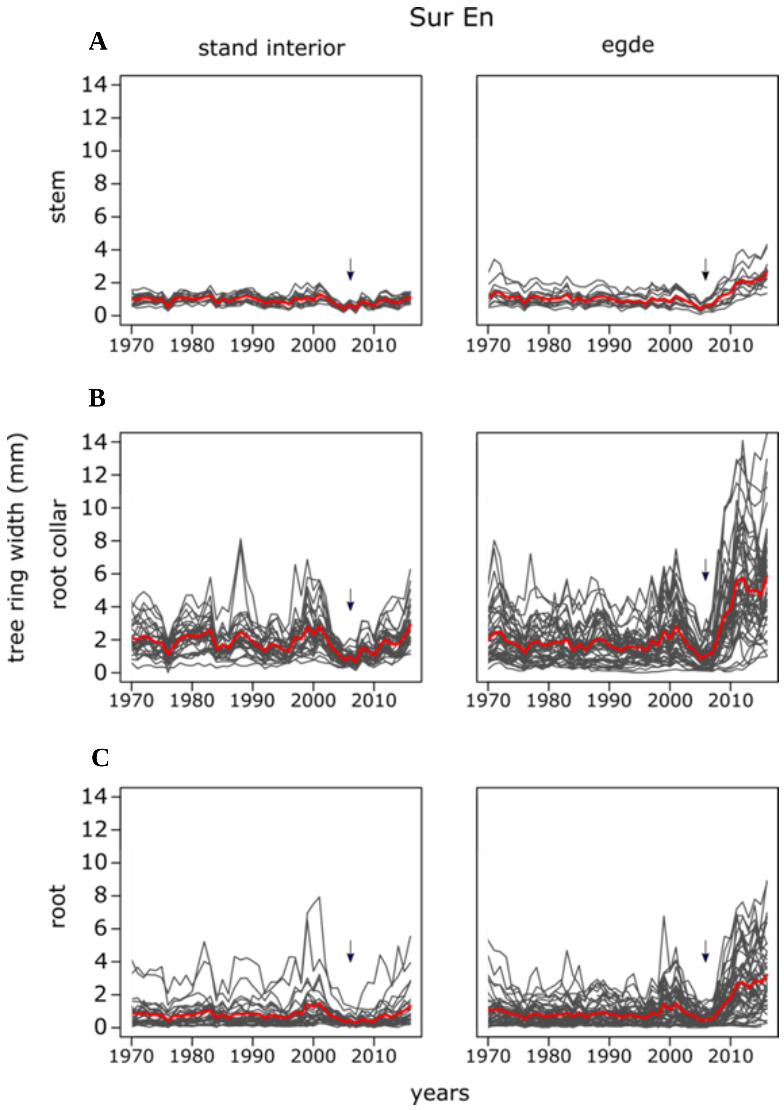

Supplement: Supplementary file 1 [file Data_Sheet_1.PDF]
